# Editorial: Polyphenols in food authenticity through the application of advanced omics technologies

**DOI:** 10.3389/fnut.2022.998635

**Published:** 2022-08-15

**Authors:** Charalampos Proestos, Mirjana Pesic

**Affiliations:** ^1^Department of Chemistry, National and Kapodistrian University of Athens, Athens, Greece; ^2^Faculty of Agriculture, University of Belgrade, Belgrade, Serbia

**Keywords:** polyphenols, authenticity, extraction, advanced omics technologies, nutrition

Polyphenols are naturally occurring bioactive compounds mainly in plant derived foods and they are attracting increasing attention in the food industry and within researcher's societies, since they have be proved to have several applications within nutraceutical, pharmaceutical, and the food industry mainly as bioactive compounds of drugs, supplements, and replacing chemical preservatives. The latter are considered to have toxic effect on humans, hence there is a tendency to replace them with plant derived compounds, such as plant polyphenols. The geographical origin and the cultivation method of these plants has been proved to be essential for the categories of compound present in plants. Many researchers have shown that some plant materials can be adulterated by others of different country origin, thus it is important to establish rapid methods to check authentication and adulteration (1). Polyphenols can serve as marker compounds and can be characteristic of ingredients and/or foods that is why many researchers are working on such projects. This Research Topic is focusing on polyphenols and how can be used as potential markers for food authentication. Not only authenticity, but also quality control of foods demand novel and hyphenated analytical techniques, omics approaches, both targeted and untargeted.

New trends in mass spectrometry for polyphenol analysis [multiple quadrupole (LC-MS*n*), time-of-flight (QTOF) LTQ-Orbitrap] include techniques such as neutral loss, precursor ion scan, and product ion scan, combined with available free online and commercial databases, give us the opportunity to detect bioactive compounds such as polyphenols even in very low amounts. Metabolomics has attracted a lot of researchers during the last 5–10 years, to work on the analysis of foods and plant derived products for authenticity and fraud issues. It is based mainly on high-resolution NMR or MS spectrometers instruments. QTOF MS is also another useful analytical technique making use of automatic acquisition of MS spectra and MS/MS in a single chromatographic injection. MS-based metabolomics by ion trap and lately the Orbitrap, delivers single and multistage mass analysis (MS*n*) for structural information. Mass measurements and elemental composition assignment are essential for the characterization of small molecules including polyphenols. GC-MS analysis is also another used technique; however, derivatization of polyphenols is needed to become volatile, and it is a semi-quantitative technique. Commercial libraries with mass spectra of standard compounds are used to complete characterization of unknown compounds. Lately, NMR spectroscopy techniques (1D HMQC, 2D diffusion-ordered spectroscopy) are preferred by researchers for identification and characterization of phenolic compounds (2).

Currently multi-omics approaches, and data analysis are implemented in the field of polyphenol studies. A combination of metabolomics, genomics, proteomics, metagenomics, and/or transcriptomics, could give answers to the field of polyphenol research (3).

Improving knowledge and understanding on polyphenols extraction and isolation from foods along with their characterization *via* modern and hyphenated analytical techniques in combination with chemometrics is the role of this Research Topic, mainly in order to discover new polyphenols that could benefit human health.

In this Research Topic, Dossou et al. by the use of LC-MS/MS they analyzed extracts of black and white sesame seeds, where they found some phenolic compounds as precursors of the sesame melanin. Reddy et al. used metabolomic approaches to characterize the extracts of plants of *Sceletium sp*. and how polyphenols present in extracts can modulate anxiety and depression. Han et al. developed oxygenated heterocyclic aglycones (OHAs) models for the authentication of different type of juices. After composing target library, quantitation was done in adulterated and non-adulterated juices. This work can be used by quality control laboratories to check adulteration in juices by implementing the fraud model used. Nedić et al. employed UHPLC-DAD-MS/MS to identify and characterize polyphenols and phenolic compounds and in combination with chemometrics as markers for geographical origin of honeys. Fotirić Akšić et al. used modern analytical techniques, to determine polyphenols in apples from Norway by implementing a UHPLC LTQ OrbiTrap MS technique.

Summarizing results the extraction, analysis and health promoting effects of polyphenols has been proven. Still, we believe that even though some very interesting works are presented in this Research Topic there is a lot of things for researchers to discover for polyphenols and omics approaches. Readers will have the opportunity to check on issues such as the extraction, the omics approaches, and health benefits of polyphenols will appear clearer to the reader and reinforce the belief that polyphenols and their presence in foods is a dynamic field for more research to be done.

## Author contributions

CP wrote the introduction and the conclusion. MP wrote the central part with comments to the cited papers and references. Both authors contributed to the article and approved the submitted version.

## Conflict of interest

The authors declare that the research was conducted in the absence of any commercial or financial relationships that could be construed as a potential conflict of interest.

## Publisher's note

All claims expressed in this article are solely those of the authors and do not necessarily represent those of their affiliated organizations, or those of the publisher, the editors and the reviewers. Any product that may be evaluated in this article, or claim that may be made by its manufacturer, is not guaranteed or endorsed by the publisher.
